# The role of the frontal cortex in memory: an investigation of the Von Restorff effect

**DOI:** 10.3389/fnhum.2014.00410

**Published:** 2014-06-27

**Authors:** Anat Elhalal, Eddy J. Davelaar, Marius Usher

**Affiliations:** ^1^Department of Psychological Sciences, Birkbeck College, University of LondonLondon, UK; ^2^School of Psychological Science and Sagol School of Neuroscience, Tel Aviv UniversityTel Aviv, Israel; ^3^Department of Experimental Psychology, University of Oxford and Wahadham College, University of OxfordOxford, UK

**Keywords:** PFC, frontal lobe, free recall, von restorff, distinctiveness, categorization, semantic memory, computational modeling

## Abstract

Evidence from neuropsychology and neuroimaging indicate that the pre-frontal cortex (PFC) plays an important role in human memory. Although frontal patients are able to form new memories, these memories appear qualitatively different from those of controls by lacking distinctiveness. Neuroimaging studies of memory indicate activation in the PFC under deep encoding conditions, and under conditions of semantic elaboration. Based on these results, we hypothesize that the PFC enhances memory by extracting differences and commonalities in the studied material. To test this hypothesis, we carried out an experimental investigation to test the relationship between the PFC-dependent factors and semantic factors associated with common and specific features of words. These experiments were performed using Free-Recall of word lists with healthy adults, exploiting the correlation between PFC function and fluid intelligence. As predicted, a correlation was found between fluid intelligence and the Von-Restorff effect (better memory for semantic isolates, e.g., isolate “cat” within category members of “fruit”). Moreover, memory for the semantic isolate was found to depend on the isolate's serial position. The isolate item tends to be recalled first, in comparison to non-isolates, suggesting that the process interacts with short term memory. These results are captured within a computational model of free recall, which includes a PFC mechanism that is sensitive to both commonality and distinctiveness, sustaining a trade-off between the two.

## Introduction

Free recall of word lists is a central experimental paradigm that has driven research on the nature of memory encoding and retrieval processes and their neural substrate (Craik and Lockhart, 1972; Tulving et al., 1994; for a review, see Davelaar and Raaijmakers, 2012). Early memory research has shown that a major factor in enhancing memory performance is the depth of encoding: one remembers more words when attending to semantic relations between the words than when attending to their sound (Craik and Lockhart, 1972). This research also showed the importance of semantic relations between list words, both in the encoding and retrieval processes. For example, many studies have demonstrated enhanced memorability for semantically related words (e.g., Glanzer and Schwartz, 1971; Greene and Crowder, 1984; Davelaar et al., 2006), and others have shown that semantic clustering takes place spontaneously in the free-recall of categorized lists (Bousfield, 1953).

A series of neuropsychological and imaging studies indicate that semantic memory enhancement involves the prefrontal cortex (Moscovitch, 1994; Tulving et al., 1994; Gershberg and Shimamura, 1995; Baldo et al., 2002; Kishiyama et al., 2009; Løvstad et al., 2012). A standardized neuropsychological test which is utilized to measure the frontal lobes' contribution to memory is the California Verbal Learning Test (CVLT-II, Delis et al., 2000). CVLT involves several memory tests including a free recall test of a categorized list. In two different studies using CVLT (Baldo et al., 2002; Alexander et al., 2003) frontal lobe patients showed semantic clustering well below healthy control participants. Converging evidence for frontal lobe involvement in semantic clustering comes from ageing studies. For example, older people produce less clustered output on the CVLT test (norm tables in Delis et al., 2000). Ageing has many shared deficits with frontal lobe impairments, and it was suggested that frontal cortex decline in elderly people is responsible for these impairments (for a detailed list: Haarmann et al., 2005).

One important strategy for utilizing the relation between words to enhance memory performance (which is central to our investigation here) is to look for distinctiveness. For example, Hunt and Lamb (2001) reported that isolate-words presented within a list of related words (all of which belong to a single category that is different from the isolate) have higher probability of recall in a free recall test. For example: an isolate word, “Hour” presented within the animal list: “Bear, Pig, Elephant, Deer, Cat, Mouse, Cow, Tiger, Horse, Lion, Rat.” This manipulation belongs to a family of experiments, usually called the “Von Restorff” (VR) paradigm, after Von Restorff who initially designed it (Von Restorff, 1933). It has also been proposed that the mechanism that mediates the effects of novelty and distinctiveness is related to frontal cortex functions (Fabiani et al., 1998; Daffner et al., 2000; Ranganath and Rainer, 2003; for a detailed overview, see Kishiyama et al., 2009).

More recently, a number of neuropsychological investigations have provided evidence to support the frontal mediation of novelty-based encoding in memory. In particular, studies by Knight and colleagues (Kishiyama et al., 2009; Løvstad et al., 2012) have shown that the prefrontal cortex modulates the Von Restorff effect. Kishiyama et al. (2009) tested 16 patients with damage to the unilateral PFC (9 Left, 7 Right) on a Von Restorff paradigm for recognition memory of object images. Whereas the age-matched control group exhibited a novelty advantage in recollection, the patients did not show any novelty advantage. In a follow up study, Løvstad et al., (2012) observed that patients with orbitofrontal or lateral prefrontal lesions exhibited a reduction in the novelty-induced P3 response over frontal electrodes. The lateral PFC patients showed sustained slow wave activity compared to the orbitofrontal patients, suggesting that the two areas are instrumental in novelty processing and are partially differentiated in their contributions. This conclusion is consistent with a number of theories that attribute several memory processes to the PFC: encoding of similarities along with distinctiveness in the material in a dynamic way (Shimamura et al., 1995; Fletcher et al., 2000; Shimamura, 2000; Frith et al., 2004).

The central aim of our study is to examine the factors that contribute to the memory enhancements related to semantic structure and semantic isolates (VR-effect), and their frontal mediation. Research on the VR-effect concentrated mainly on the improved memory of items that are “physically” distinctive from other items in the experiment (different in color, sound, font, etc). However, there is evidence that somewhat different processes are involved in the processing of semantic and physical distinctiveness (Fabiani and Donchin, 1995). Surprisingly few experiments have investigated the nature of semantic Von-Restorff effect, and specifically the effect of the serial position of an isolate word in free recall memory tests on its enhanced memorability. One would expect the isolate advantage in free recall to depend on the serial position of the isolate in the list. In the extreme case, when the isolate is the first item in the list, it is still not distinctive as the participant does not know what comes next. In later serial positions, a few related words have already been presented and thus the isolate word is more distinctive.

We start with an experimental investigation of the correlations between a number of semantic effects in memory recall (in particular VR and semantic clustering) and fluid-intelligence—a measure associated with frontal function. The results indicate the presence of individual differences (fluid-intelligence), previously associated with the lobes (Duncan et al., 1995, 1996, 2000) that mediate both VR and clustering effects. As both temporal clustering (CVLT) and novelty based encoding were found to be deficient in patients with frontal lesions, we will take it as our working hypothesis that they are both mediated by a frontal mechanism. This hypothesis is obviously tentative at this stage (see General Discussion). Based on this, we developed a neurocomputational model that makes specific parametric predictions for VR-effects and semantic clustering in free recall. Finally, two experiments were carried out that confirmed these predictions.

## Experiment 1: a correlational study of the relationship between fluid intelligence and semantic factors in free recall

Experiment 1 was designed to explore the role of the frontal cortex in semantically related memory functions. In order to investigate this with healthy young adults, a correlational approach was adopted. A correlation between frontal cortex activity and performance on fluid intelligence tests was previously demonstrated in tasks such as the Cattell Culture Fair Test (Duncan et al., 1995, 1996, 2000). Therefore, participants' performance in this test was measured and translated into IQ scores. The participants carried out a free recall test of different types of lists to measure memory components which are believed to be related to the frontal cortex. Types of lists included semantic isolates (Von Restorff), lists of categorized words, lists from the DRM paradigm, and lists designed to measure proactive interference (PI).

Investigations of frontal patients show that semantic clustering is mediated by the frontal cortex, as frontal lobe patients frequently show semantic clustering well below healthy control participants (Gershberg and Shimamura, 1995; Baldo et al., 2002). In the DRM paradigm (Deese, 1959; Roediger and McDermott, 1995), participants are presented with lists of words, all strong associates of a non-presented target word^1^. False memory for the target word is measured. Melo et al. (1999) showed initial evidence of frontal patients producing more false memories than controls on the DRM paradigm, in agreement with the hypothesis that relates utilizing common features between words in memory to PFC functions. It was previously found that frontal patients show enhanced sensitivity to proactive interference (for example, Shimamura et al., 1995). However, other scholars have claimed that it is the release from proactive interference that is the main deficit in frontal patients (Moscovitch, 1982).

### Methods

#### Participants

The study was carried out as part of an undergraduate practical class, in which students participate in an experiment in order to produce their own empirical data and subsequently analyze it. Eighty-one students at Birkbeck College took part in the experiment, ages ranging 21–50, with a mean of 32. Since the focus of the experiment was semantic effects, non-native English speakers were excluded from the sample. Thirty-two participants did not meet the criteria of living in English speaking countries from the age of 12 or younger. Two more participants were excluded, since their measured IQ score was 60. This score was assumed to not represent the true IQ of university students, indicating they did not engage with the test truly. Eventually, data was analyzed from the remaining 47 participants.

#### Design

This experiment was of a correlational design. Measured variables were IQ and 5 different types of semantic memory scores.

#### Materials

The Cattell test was used to measure IQ.

Thirty lists of 12–15 words each were used to measure specific memory functions. All lists involved semantically related words. Categories were chosen to have no overlap to minimize between-lists effects. All categorized lists (except those of the original DRM task) were created using the Van Overschelde et al. (2004) norms.

***DRM lists***. Six lists were taken from the DRM paradigm (Roediger and McDermott, 1995) from which 15 words were presented; these were all strong associates of a word that was not presented. For example, the words: *hot, snow, warm, winter, ice, wet, frigid, chilly, heat, weather, freeze, air, shiver, arctic, frost* were presented, which are all associated with the word *cold* which was not presented. The number of times the non-presented words were falsely recalled was counted. The following DRM lists were used: feelings, colors, food, furniture, weather, medicine (from Roediger and McDermott, 1995).

***Von Restorff lists***. Six categories were chosen from the Van Overschelde et al. (2004) norms. Lists of 11 words were created from each category, with the 12th word from an unrelated category inserted in different positions in the list, as the 1st, 6th, and 10th word. For example: Beer, Vodka, Rum, Whiskey, Tequila, Gin, Liquor, Scotch, Martini, House, Bourbon, Daiquiri.

***Categorized words lists—blocked***. Six lists of 12 words were created, with words taken from three different categories (from the Van Overschelde et al. (2004) norms, four words from each category). First the words from the first category were presented, then from the second, then third. For example: Eagle, Robin, Hawk, Crow, **Priest, Pope, Bishop, Nun**, *Barbie, Ball, Puzzle, Lego*.

***Categorized words lists—cyclical***. Six lists of 12 words were created, with words taken from three different categories (from the Van Overschelde et al. (2004)norms, four words from each category). First, a single word from the first category was presented, then a single word from the second, then from the third, and so on. For example: Rose, **Ballet**, *Window*, Tulip, **Tango**, *Door*, Lily, **Salsa**, *Wall*, Iris, **Waltz**, *Floor*.

***Proactive interference lists***. Six lists of 12 words were constructed. Every two consecutive lists were with words from the same category.

The memory test was created using the Eprime environment for psychological testing (pack 1.1).

#### Procedure

The experiment was carried out in a classroom. Participants were instructed to keep silence at all times to avoid interfering with other participants. Participants first performed the Cattell test, followed by the memory test. Participants were told they would be presented with lists of words which they should read subvocally, and thereafter try to remember as many of them as possible in any order.

The memory test comprised of 30 lists of words. Each word was presented for 1.5 s. After each list a recall box appeared and participants typed in as many words as they remembered. Instructions were to press “Enter” after every word, which cleared the recall box for a new word. Pressing “Enter” on an empty box was the agreed sign for no more recalled words. That led to a screen asking them to “press any key to move to the next list.” Participants were given 60 s to recall words, after which the program moved to the same screen automatically. Participants were first briefed with this procedure by a class presentation which was followed by individual practice prior to the experiment. The whole experiment took under 1 h, with the Cattell test lasting about 25 mins (including instructions). Participants then moved to a computer room and proceeded with the memory test for 30 min.

### Results

IQ scores were calculated based on Cattell test results. Mean IQ for the sample was 107, with a standard deviation of 13. This is above the population mean, as one would expect from a sample of university students.

#### Memory for isolate words (von restorff effects)

**Figure 1:**
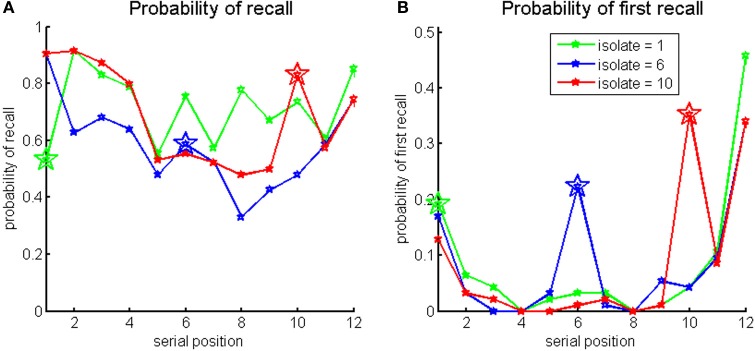
**Probabilities of recall (A) and first recall (B) for the Von Restorff lists by the isolate serial position in the list: green for 1, blue for 6 and red for 10**.

IQ was correlated with the total number of words recalled from VR lists: *r*_(45)_ = 0.335, *p* < 0.05. In addition, IQ was correlated with different measures of the isolation effect. Correlation between IQ and the number of isolates recalled was *r*_(45)_ = 0.407, *p* < 0.005. To show that the impact of IQ is specific for the isolates, the number of isolates recalled was divided by the total number of list-words recalled. The resulting measure was correlated with IQ as well: *r*_(45)_ = 0.336, *p* < 0.05. Isolates appeared in three serial positions in VR lists: 1st, 6th, and 10th. Looking for the number of isolates produced from each serial position separately, only the correlation with the 6th position (divided by the total number of words recalled) reached significance: *r*_(45)_ = 0.403, *p* < 0.005. For the 1st and 10th positions, correlations were lower and non-significant [for the 1st: *r*_(45)_ = 0.198, *p* = 0.181, NS; for the 10th: *r*_(45)_ = −0.32, *p* = 0.828, NS]. A further measure of the VR-effect was calculated: the number of times a word is recalled from each of the three serial positions when it is an isolate, compared to when it is not an isolate (this could be found from the other VR lists in which isolates appeared in different serial positions). Significant correlation was found again, for the 6th serial position only: *r*_(45)_ = 0.303, *p* < 0.05 [for the 1st: *r*_(45)_ = 0.197, *p* = 0.185, NS, for the 10th: *r*_(45)_ = −0.068, *p* = 0.651, NS]. Probabilities of recall and first-recall are presented in Figure 1.

**Figure 2:**
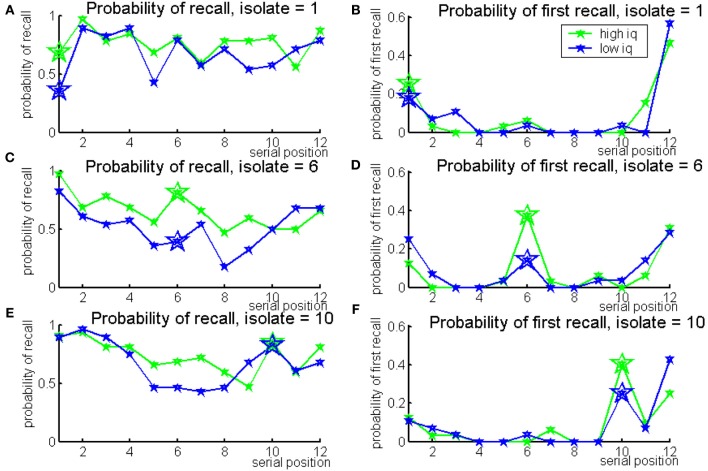
**Probabilities of recall (left panels) and first recall (right panels), for the Von Restorff lists by the isolate serial position in the list, split by IQ median score (green for higher-IQ individuals, blue for lower-IQ individuals)**.

The results show higher probability of recall for isolate items at late serial positions, and higher probabilities of first recall for all serial positions tested. Serial position curves exhibit high noise. This could be attributed to the experimental design which was optimized for correlation analysis, and hence there was no counterbalancing or randomization in lists presentation. In addition, only two lists were presented per participant per isolate serial position which may have contaminated the data with word specific effects. The probabilities of recall and first recall split by the IQ median score are presented in Figure 2. It can be seen that higher-IQ individuals tend to show more advantage for the isolates in comparison to lower-IQ individuals, as verified by the correlation analysis.

#### Memory for categorized lists: blocked and non-blocked

IQ was correlated with the total number of words recalled from categorized lists: *r*_(45)_ = 0.335, *p* < 0.05. In addition, IQ was correlated with different measures of categorization. Categorization factors were calculated in accordance with the CVLT test method (Stricker et al., 2002). Essentially, the number of pairs of same category words that were recalled consecutively was counted and corrected by the number of such pairs expected to occur by chance. High correlations with IQ were found both for the blocked lists [*r*_(45)_ = 0.464, *p* < 0.05] and the non-blocked lists [*r*_(45)_ = 0.367, *p* < 0.05].

**Figure 3:**
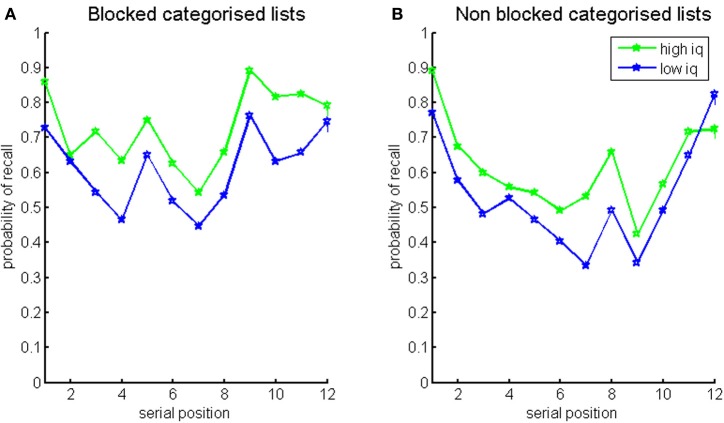
**Probabilities of recall for blocked categorized lists (left) and non-blocked categorized lists (right), split by IQ median score (green for higher-IQ individuals, blue for lower-IQ individuals)**.

Probabilities of recall for the blocked and non-blocked lists of categorized words split by the median IQ score are presented in Figure 3. This shows an overall advantage for higher-IQ individuals in both conditions (also see Supplement, Figure S1 for a first recall probability, showing that High-IQ participants have an increases tendency to start recall with the first item of the last category—a novelty effect).

Counter to predictions, no correlations were found with any measures of the proactive interference lists, nor the DRM lists. This could be due to the homogeneity of the experimental sample, which may have produced less differentiation than between healthy subjects and patients.

Finally, we report in the Supplement (Figure S2) temporal order effects (lag-CRP; Howard and Kahana, 2002) for randomized and categorized lists (see Discussion).

### Discussion

Correlations were found between fluid-IQ and semantic clustering, as well as IQ and the Von Restorff effect for mid-list items. The correlation between IQ and semantic clustering in healthy adults supports similar results found in frontal patients and neuroimaging (Gershberg and Shimamura, 1995; Savage et al., 2001; Baldo et al., 2002). It was expected that a relationship would be established between the PFC and sensitivity to novelty, as well as distinctiveness. This is based on results from other paradigms (Ranganath and Rainer, 2003; Kishiyama et al., 2009; Løvstad et al., 2012), and supported by the newly found correlation between the Von Restorff effect and frontal functions (for mid-list isolates). In agreement with patients and neuroimaging data, correlations were also found between semantic clustering and IQ. Counter to predictions, however, no correlations were found with proactive interference or release from proactive interference.

In order to better understand the mechanism by which the PFC utilizes the relationship between words in a list to enhance both semantic clustering and the memory of semantic isolates, we developed (in the next section) a computational model. This was based on the activation-buffer model that was previously used to account for data in immediate-free-recall (IFR) and in the continuous distractor task, and the dissociations between them (Davelaar et al., 2005, 2006).

Like in SAM (Raaijmakers and Shiffrin, 1980), this model includes a short-term activation buffer, so that the last words in the list would still be active in such a buffer at the time of recall. The frontal mechanism interacts with the buffer function to enhance encoding of semantic relationships between words. The neuropsychological literature described above and the experimental results presented here support the assumption that there are additional memory processes to those implementing the buffer which are mediated by the PFC. While the buffer only maintains some information about previously presented items in an active state, these additional mechanisms play a role in enhancing the encoding of semantic relationships (both similarities and differences) as illustrated by semantic clustering and the VR-effect. We label this model the “categorization-activation-novelty model” (CAN) to reflect the functional role of its components.

The present model does not address one important aspect of memory encoding and retrieval: the changing context. This has been demonstrated to be critical in accounting for order effects in free recall paradigms at different temporal scales (Howard and Kahana, 2002; Polyn and Kahana, 2008; Polyn et al., 2009); see also Figure S2 (in supplement) for data from Experiment 1, showing an interaction between lag-recency and semantic relation). This important component will need to be integrated into the present model in future studies (see General Discussion).

## Simulation study: the *can*-model for the role of the PFC in memory

We assume that the frontal mechanism has two main components: a fast learning categorization layer and a novelty detection mechanism (see also Grossberg, 1978a,b). The CAN model is comprised of four components: the frontal mechanism, a short term activation buffer, a layer of semantic features and a context mechanism.

### Activation buffer and the semantic layer

**Figure 4:**
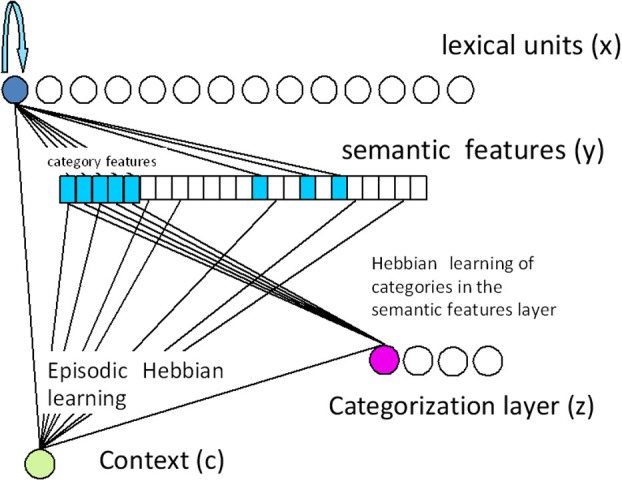
**Schematic diagram of the encoding stage of the model**. The top layer is the lexical layer, the middle is the semantic features layer. Context is represented by a single unit (list context) in the bottom-left, and the categorization layer (component of the PFC) is illustrated in the bottom-right. During list presentation, lexical units receive input. Activation spreads to the semantic features layer and further to the categorization layer. Episodic encoding by Hebbian learning takes place between the context and the lexical, semantic and categorization layers. In addition, fast Hebbian learning takes place between the categorization layer and the semantic layer.

The activation buffer is a lexical layer (each unit corresponds to a word), with recurrent excitation and global lateral inhibition (Haarmann and Usher, 2001; Davelaar et al., 2005). This leads to 3–4 items being co-active during the list presentation, allowing the model to account for serial position functions (both recency and primacy) in IFR (Davelaar et al., 2005). Since we focused here on semantic relations between the words in the list, we included for each word a semantic representation in a semantic layer whose units correspond to the semantic features of the word. Each lexical unit is thus connected with the semantic units that correspond to its “semantic representation” (see Figure 4). Words from the same category were assumed to have shared semantic representations. Finally, we assumed the existence of a list-context representation (Anderson and Bower, 1972). During list presentation, this representation becomes connected with the activated units in the semantic and the lexical layers.

### Categorization layer

The categorization layer is comprised of multiple units with strong competition and initial random connectivity to the semantic features. During the encoding phase, when a member of a certain category gets input, its semantic representation in the semantic layer becomes active. The activation sent to the categorization layer causes a categorization unit (or a few units in some cases) to win the competition in this layer. Hebbian learning then takes place between the semantic features layer and the categorization unit, hence the categorization unit is dynamically learning a new category online. The more category members presented in the list, the stronger the connections become between the category's shared features and the categorization unit. However, if an item from a different category is presented, its features activate a different categorization unit (due to the initial random connections). The new categorization unit then learns the second category. At retrieval, activation sent from the categorization layer to the semantic features layer enhances recall of category members consecutively, leading to semantic clustering.

### Novelty detection and encoding

A second frontal component detects novelty in the input by comparing the predicted (a leaky integrated value of its history; see Detailed Description) activation values in the semantic features layer to the current ones. When a large difference is detected, a “surprise” signal is produced by the novelty mechanism which boosts both the effect of the sensory input (this can be viewed as enhanced attention) and the learning rate between the categorization layer and the semantic features layer. This mechanism enables simulation of the Von-Restorff effect through the stronger encoding of an isolate. It also enables simulating the dependency of the Von-Restorff effect on the isolate's serial position as found in the experiment above. When the isolate is in the first serial position, there is still no prediction, and therefore no advantage in encoding. When a few category members have already been presented before the isolate, the frontal mechanism detects the isolate's novelty and enhances activation and learning. Therefore, the isolate can be more easily encoded and later recalled. It is predicted that the closer the isolate is to the end of the list, the higher the probability that its enhanced activation would enable it to survive the competition in the lexical layer until the end of the simulation. Therefore, isolates nearer the end of the list have a better encoding advantage.

The combination of the categorization and novelty detection mechanisms enables the model to simulate differences in semantic clustering between frontal patients and healthy controls, as well as the PFC sensitivity to novelty, the dependency of the Von-Restorff effect on the serial position of the isolate and the correlation found between PFC function and the magnitude of Von-Restorff effect. The model components are illustrated in Figure 4.

### Retrieval

At the retrieval stage, activation is sent from the context to the lexical, semantic and categorization layers. Every item that crosses the retrieval threshold is retrieved and subsequently inhibited. The PFC has an additional role in monitoring retrieval: the categorization layer is reset following the occurrence of a certain number of unsuccessful retrieval attempts.

### Detailed description

#### Encoding

Encoding a list of words is simulated by activating lexical units one at a time by an input. Activation spreads from the lexical units to the semantic features layer through a predefined connectivity matrix. Activation then spreads from the semantic features layer to the categorization layer through random initial connections. Connections between categorization units (which are active above a learning threshold) and active semantic features are strengthened. In addition, the activation in the semantic features layer is constrained by an adaptation component. When novel input is presented, the difference between the semantic features' leaky integrated value (or activation history, which is actually the adaptation component value) and the current activation is likely to cross a threshold. As a consequence, the input to the lexical layer (as well as the learning rate between the categorization layer and the semantic features) is increased (the “surprise” effect).

The model is applied to simulate free recall of categorized lists, of normal subjects and frontal patients. At the encoding stage, 12 lexical units get activated (or 16 in a CVLT simulation) for T time steps each. The following events take place at each time-step:

– The adaptation of the semantic features layer is updated
– If the difference between the semantic layer activation to the adaptation value (absolute value) crosses a threshold, the input to the lexical items as well as the learning between the semantic features and the categorization layers are raised for the current item
– The lexical layer activation is updated
– The semantic features layer activation is updated
– The categorization layer activation is updated
– The connections between the three layers: lexical, semantic features and categorization to the context, are updated
– If the activation of one (or more) categorization unit(s) crosses the learning threshold, the connections between this unit(s) and the semantic features are strengthened

***The lexical layer (activation buffer)***. The lexical layer is comprised of 50 units, out of which 12 serve as list items. During a simulation of list presentation, lexical units are activated sequentially by clamping each unit for a “sensory” input for T time steps. Units are connected to themselves via self excitation to maintain activity after input offset. They are also negatively connected to all other units in the layer which creates global inhibition. The balance between the magnitude of the self excitation and global inhibition creates a limited capacity buffer. Units that are active above threshold in the lexical layer are considered to be the short term activation buffer. When a new item is activated, its activation causes the activation of one of the previous item to diminish through the global inhibition. The new item would eventually replace the weakest item in the buffer. Buffer dynamics are described by the following equation:

$$\begin{array}{l} {\text{x}}_{\text{i}}(\text{t})=\lambda {\text{x}}_{\text{i}}(\text{t}-1)+(1-\lambda )[\alpha \text{F}\left({\text{x}}_{\text{i}}(\text{t})\right)-\beta_{\text{x}}\Sigma \text{F}\left({\text{x}}_{\text{j}}(\text{t}-1)\right)\\ \;+{\text{I}}_{\text{i}}(\text{t})+\xi \Sigma {\text{W}}_{\text{ij}}^{\text{xy}}\text{F}\left({\text{y}}_{\text{j}}(\text{t}-1)\right)+\text{N}(0,0.2)] \end{array}$$

**Figure 5:**
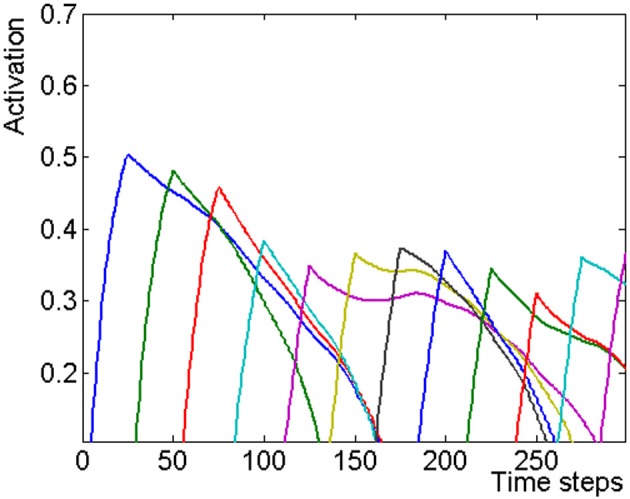
**The dynamics of the lexical layer during a 12 items list simulation**. The activation of each item is shown as a different color graph. It can be seen that the first three activated items reach higher activation values, because they face less competition from previously presented items.

Where **F(x)** = x/(1 + x) for x > 0, and 0 otherwise (O'Reilly and Munakata, 2000). **x_i_(t)**: activation of lexical item i at time t. All activations are set to zero at the beginning of a simulation. **λ**: a time constant, controlling the amount of change in each time step; **α**: the self-excitation parameter; **β**_x_: the global inhibition parameter; **I_i_(t)**: the sensory input to unit i at time t. The input is clamped to each list unit for **T** time steps. Each lexical unit gets activation from the semantic layer: **ξΣW^xy^_ij_F(y_j_(t − 1))**. The activations of the semantic layer are denoted by **y_j_**. **W^xy^** is the connectivity matrix, (the superscript **xy** stands for the connections from the semantic features, **y**, to the lexical items, **x**, all connections are symmetric) and **ξ** is the parameter multiplying that activation. The activation in the layer is not allowed to get negative. The activation of the lexical units during a presentation of a list of 12 words is shown in Figure 5.

***The semantic features layer***. The semantic features layer is comprised of 45 units which are reciprocally connected with the lexical units through the connectivity matrix **W^yx^**. Competition in the semantic features layer is mediated by global inhibition, similar to the lexical layer. Semantic features receive activation from the lexical layer (through **W^yx^**), as well as input from the frontal mechanism (categorization layer and the “surprise” signal). The semantic features activation is described by the following equation:

$$\begin{array}{l} {\text{y}}_{\text{i}}(\text{t})=\lambda {\text{y}}_{\text{i}}(\text{t}-1)+(1-\lambda )[-\beta_{\text{y}}\Sigma \text{F}\left({\text{y}}_{\text{j}}(\text{t}-1)\right)\\ \;+\chi \Sigma {\text{W}}_{\text{ij}}^{\text{yx}}{}^{\prime }\text{F}\left({\text{x}}_{\text{j}}(\text{t}-1)\right)-\kappa {\text{a}}_{\text{i}}(\text{t})] \end{array}$$

Where **y_i_(t)** is the activation of the semantic feature i at time t, **λ** and **F(y)** are the same as described above, **β_y_** is the global inhibition parameter and **χΣW^yx^_ij_F(x_j_(t - 1))** is the activation the semantic features receive from the lexical layer.

The term **a_i_** represents the adaptation in the semantic features layer.

Its value is updated in every time step, according to the semantic features activation:

$${\text{a}}_{\text{i}}(\text{t})=\lambda_{\text{a}}{\text{a}}_{\text{i}}(\text{t}-1)+(1-\lambda_{\text{a}})\text{F}\left({\text{y}}_{\text{i}}(\text{t}-1)\right)$$

In addition, the activation in the layer is not allowed to get negative.

***The connectivity matrix between the lexical and the semantic layers***. Each lexical unit is connected via the connectivity matrix **W^xy^** with a number of semantic units. These connections are symmetric: **W^xy^_ij_ = W^yx^_ji_**. Since the lexical layer corresponds to a localistic representation of items, the pattern of **W^xy^** corresponds to a distributed representation of each item in terms of semantic features. The connectivity matrix between the lexical items layer and the semantic features layer is as follows: **W^xy^** is defined in the beginning of each simulation. To simulate a list of unrelated items, each element of the matrix is set to one with a probability of 16%, which means that on average each lexical unit is represented by 6–7 semantic features. To simulate a list of words which belong to the same semantic category, a set of features (usually 5) is set to one for all of these words, in addition to randomly selected distinctive features. See Figure S3 (in the supplement) for a connectivity matrix of a lexicon in which words belong to 4 different categories which are represented by sets of 5 features each.

The following two model components are the categorization mechanism and the novelty detection mechanism, which we associate with the PFC.

***The categorization mechanism***. The categorization layer consists of 10 units. Their activation is initialized to zero at the beginning of the simulation. The dynamics of the categorization units' activation is described by the following equation:

$$\begin{array}{l} {\text{z}}_{\text{i}}(\text{t})=\lambda {\text{z}}_{\text{i}}(\text{t}-1)+(1-\lambda )[\text{​}-\beta_{\text{Z}}\Sigma \text{F}\left({\text{z}}_{\text{i}}(\text{t}-1)\right)\\ \;+\phi \Sigma {\text{W}}_{\text{ij}}^{\text{zy}}\text{F}\left({\text{z}}_{\text{j}}(\text{t}-1)\right)] \end{array}$$

Where **z_i_(t)** is the activation of categorization unit i at time t, **λ** is a time constant, controlling the amount of change in each time step, **β_Z_** is the global inhibition parameter. **ϕΣW^zy^_ij_F(z_j_(t − 1))** is the activation sent from the semantic features layer, **W^zy^** is the connectivity matrix between the categorization and the semantic features layer.

The categorization layer is reciprocally connected with the semantic features layer. The connections are initialized to random values, distributed between 0–1: W^yz^(t = 1) ϵ U(0,1). Hebbian learning takes place between categorization units which cross a learning threshold and the semantic features:

$${\text{W}}^{\text{yz}}={\text{W}}^{\text{yz}}+\delta \text{F}{\left(\text{z}\right)}^{\text{T}}\text{F}(\text{y})$$

The connections between each categorization unit and all features are normalized after each update.

***The novelty (distinctiveness) mechanism: enhancing attention and encoding of novel stimuli***. At each time step, the novelty mechanism is monitoring the difference between the current activation in the semantic features layer and the predicted one, which is mathematically identical to the definition of the adaptation term: **a(t)**. If this difference crosses a threshold, the attention to stimuli (or studied material) is enhanced by increasing the input parameter (to the lexical items layer) to **I_s_**. In addition, the learning between the categorization layer and the semantic features layer is increased to **δ_s_**.

When simulating a frontal patient, no learning takes place between the categorization layer and the semantic features layer, and stimulus novelty is not detected.

***The context “layer.”*** Context is represented by a single unit in this model, and can be seen as a “list context.” Episodic Hebbian learning takes place between the context and the lexical items layer, the semantic features layer and the categorization layer:

$$\begin{array}{l} Cx_{i}=Cx_{i}+F(x_{i}(t))\\ Cy_{i}=Cy_{i}+F(y_{i}(t))\\ Cz_{i}=Cz_{i}+F(z_{i}(t)) \end{array}$$

Where **Cx**, **Cy,** and **Cz** are the (vectors of) magnitudes of the connections of the context to the different layers, respectively.

#### Retrieval

At retrieval, the categorization layer activates the semantic features layer. In addition, the context activates the semantic features, the lexical items and the categorization layers. When the activation of a lexical item crosses a retrieval threshold, it is considered to be recalled. Its activation is reset after recall. If more than 2 unsuccessful retrievals have been made (repetitions and intrusions counted together), the activation of the categorization layer is reset.

The following steps take place for **TR** time steps:

– The adaptation of the semantic features layer is updated
– The lexical layer activation is updated
– The semantic features layer activation is updated
– The categorization layer activation is updated
– If the activation of a lexical unit crosses the retrieval threshold, it is considered to be recalled (and following reset)
– If more than two unsuccessful retrievals occurred, the categorization layer is reset

***The lexical layer (activation buffer)***. The dynamics of the lexical layer in retrieval is similar to encoding, aside from the activation received from the context. The term **μ_x_Cx_i_(t − 1)** is added to the equation.

***The semantic features layer***. Two additional terms are added to the dynamics of the semantic features layer in retrieval: the activation received from the context **(μ_y_Cy_i_(t − 1))** and that received from the categorization layer **(ϖΣW^yz^_ij_F(y_j_))**. When a lexical item's activation exceeds the retrieval threshold, it is considered to be recalled. A recalled item can be a successful one if a list item is recalled for the first time, or an unsuccessful one when an item is repeated, or when an item which was not activated during encoding becomes active during retrieval (intrusion). When an item is recalled its activation is inhibited.

***The categorization layer (and connectivity matrix to the semantic features layer)***. The dynamics of the categorization layer in retrieval is similar to encoding, aside from the activation received from the context. The term **μ_z_Cz_i_(t − 1)** is added to the equation. Furthermore, the value of the parameter multiplying the activation received from the semantic features layer (**ϕ**) is reduced. It is more important for the PFC to be affected by successful/unsuccessful retrievals, than by the current activation of the semantic features layer.

In addition to the described dynamics, the unsuccessful retrievals are monitored. This is modeled as a symbolic algorithm rather than a neural network since this process is outside the scope of the current modeling attempt. When the number of unsuccessful retrievals (repetitions or intrusions) exceeds 2, the activation of the categorization unit(s) that were active during the recalls are inhibited for the rest of the simulation. In addition, the activity of the whole categorization layer is reset. A different categorization unit will become active due to the activation received from the context.

### Simulation results

The model was applied to simulate semantic clustering in free recall as well as the experimental Von-Restorff results presented earlier. In particular, the difference between frontal patients and normal subjects is explored. In order to simulate a frontal patient, the frontal mechanisms are disabled, hence no learning takes place between the categorization layer and the semantic features layer and novelty is not monitored.

#### Modeling clustering of a categorized list

As part of the CVLT task (Delis et al., 2000) participants are asked to memorize a list of 16 words from four different categories which are presented in a random order. The CAN model is used to simulate the differences in semantic clustering between frontal patients and normal subjects. The CVLT includes a few repetitions of encoding and retrieval of the list. Only the first trial is simulated here.

In the simulation, the lexicon for this task is constructed of four categories, with four items in each category. These 16 items are presented in a random order with no two consecutive items from the same category, similar to the CVLT paradigm. Presentation order changes with every run of the simulation (see Figure S3 in Supplement). In the Supplement we show a detailed illustration of the semantic representations of list items and the operation of the categorization units during encoding and retrieval.

**Figure 6:**
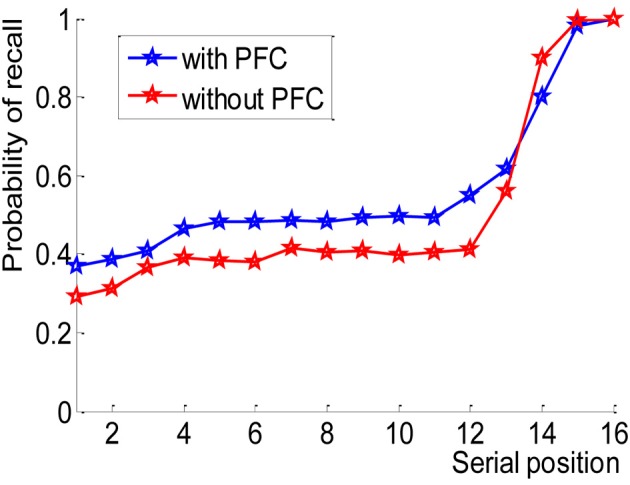
**Probability of recall of a CVLT—like list, red: model without the frontal mechanisms, blue: model which includes the frontal mechanisms**. The frontal mechanisms enhance recall of a categorized list.

Both the model without the frontal mechanisms and the one which included them were able to recall the CVLT–like lists (see Figure 6), the latter remembering consistently more words, except in the recency positions which are mediated by the lexical buffer. In the model without the frontal mechanisms, recency is due to the items which were still active in the lexical layer at the time of recall. Adding the frontal mechanisms created preference for category members of the most active items, lowering the recall of item number 14.

Semantic clustering index was calculated in a similar way to the CVLT test (Delis et al., 2000; Stricker et al., 2002). The model without the frontal mechanisms output was less categorized than the model with the frontal mechanisms: 2.8 (±1.8) in comparison with 5.4 (±2.2) (*p* < 0.001). For example, in a single trial in which the presented list was: a1, d1, c1, d2, c2, d3, a2, b1, a3, b2, c3, a4, b3, d4, b4, c4 (letters represent categories, numbers represent category exemplars), The model with the frontal mechanism produced the following output: b4, c4, d4, c2, c4, c3, d3, d2, d4, c2, d3, a2, a1 (The first recalled items are the recency items, therefore are not categorized). Alternatively, the model without the frontal mechanisms produced: c4, b4, d4, c3, a4, a1, c4, d3, d1.

#### Modeling the VR-effect

The experimental results indicate that while isolate items have advantage in late serial positions, they are less well remembered when they appear in the beginning of the list. The CAN model is applied to simulating these data. The lexicon for this task is constructed of two categories. All studied items except one belong to a single category, while the isolate item belongs to a different category. See the supplement for detailed illustrations of the encoding and retrieval phases.

**Figure 7:**
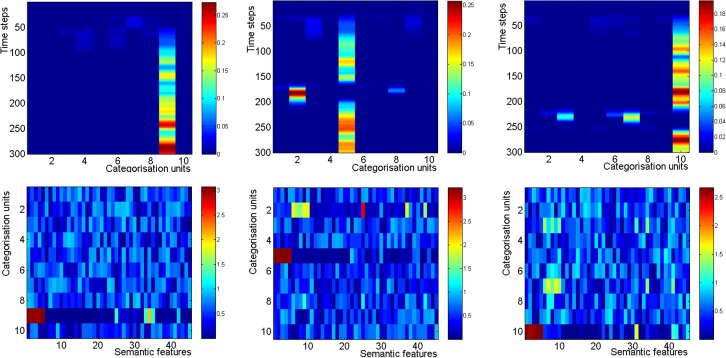
**The categorization layer in a Von Restorff simulation**. In both panels, the isolate appears in serial position 1 (left) 7 (middle) and 9 (right). An isolate at position 7 gets activated during time steps 150–175, and an isolate in serial position 9 gets activated during time steps 200–225. Warmer colors represent higher activations. **Top**: The activation in the categorization layer, in a simulation which includes the frontal mechanisms. **Bottom**: The connectivity matrix between the semantic features and the categorization layer. It can be seen that a categorization unit gets associated with semantic features 1–5 (which represent the first category) and a different categorization unit gets associated with semantic features 6–10 (which represent the isolate category).

During the presentation of a category, a categorization unit becomes associated with it. When an isolate is activated, usually a different categorization unit (different from the one that learned the category of the other list items) becomes active and becomes associated with the isolate. The items activated after the isolate belong to the first category which was already learned by the former categorization unit. That categorization unit becomes reactivated due to the learning that already took place before the isolate presentation. The activation in the categorization layer can be seen in the top panel of Figure 7. For example, in the middle panel the isolate appears in serial position 7 (at around *t* = 180). Categorization unit number 5 gets associated with the main category, while categorization unit number 2 (and 8 to some extent) get associated with the isolate.

The learning which is taking place between the categorization layer and the semantic layer is demonstrated by the changes in the connectivity matrix between them (see bottom panel of Figure 7). It can be seen that different categorization units become associated with the main category items and the isolate item, except when the isolate appears in serial position 1.

**Figure 8:**
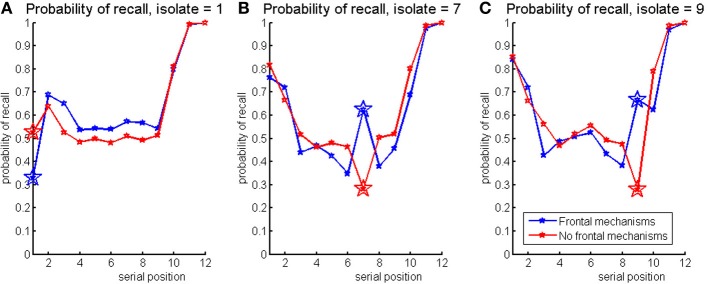
**Probabilities of recall for the model without (red) and with (blue) the frontal mechanisms, simulating Von-Restorff lists with isolate in the 1st (left) 7th (middle), and 9th (right) position**. It can be seen that the isolate is better recalled in the model with the PFC mechanism and this advantage depends on its serial position as predicted.

The model was used to simulate the Von-Restorff paradigm with isolates in the 1st, 7th, and 9th serial positions. Serial position curves are presented at Figure 8 for the model without (red) and with (blue) the frontal mechanisms. The frontal mechanisms make it possible to remember the isolates. The advantage of the isolate is larger, closer to the end of the list as found in experiment 1. Without the frontal mechanisms the isolate is poorly remembered at all serial positions. These results are a good qualitative fit to the experimental results, especially the relative difference between the two simulations. However, the model results show some advantage of the isolate in serial position number 7 (Von Restorff effect) which is not present in the data.

#### Modeling the von restorff effect—additional baseline

The CAN model replicates the well known result that words in semantically related lists have higher probability of recall, than words in unrelated lists (for example, Glanzer and Schwartz, 1971; Greene and Crowder, 1984, and more recently, Davelaar et al., 2006). If words from a semantically related list are better remembered, a prediction could be made for disadvantage for the isolate item. We have modeled the higher probability of recall for an isolate, or the Von Restorff effect, as a “surprise,” or novelty/distinctiveness signal which enhances memorability. This advantage however, is predicted to depend on the serial position within the list as at the start of the list, the isolate is still not distinctive. It can thus be expected that in earlier serial positions (especially if first in the list) the isolate is less likely to be retrieved, but in later serial positions (especially close to the end of the list, i.e., recency items) this tendency is inverted and gives the isolate a better probability of recall.

In previous experiments with semantic isolates, memory for an isolate word was compared to memory of a word of the same category as the rest of the list, in the same serial position (e.g., Hunt and Lamb, 2001).

**Figure 9:**
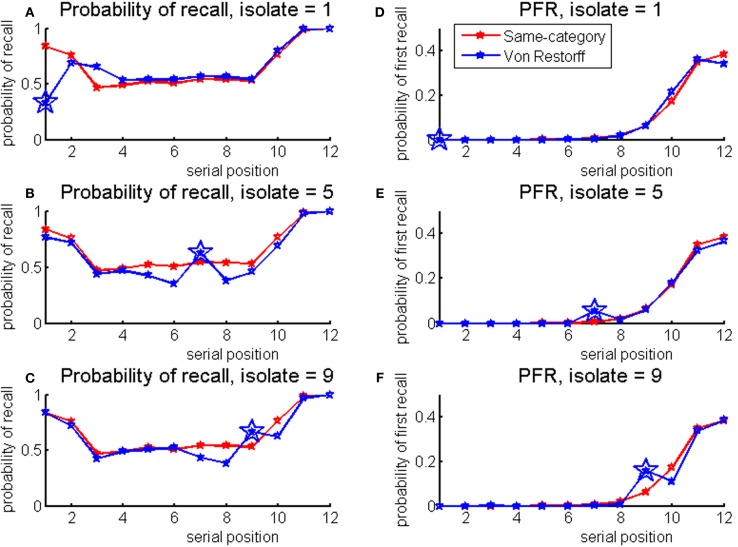
**Probabilities of recall and first recall for the model with the frontal mechanisms for Von-Restorff simulations, with isolates at the 1st (left) 7th (middle) and 9th (right) serial positions**. Blue: Von-Restorff list, Red: same-category list baseline.

To simulate a same-category list, semantic features 1–5 were selected for all items. Results are presented in Figure 9.

First, the simulation shows that isolate items (star on blue lines) are better remembered than their within list neighbors and than the same word in a same-category list, but only at middle and recency position (this can be seen in both total recall, Figure 9 left, and in first recall probability, Figure 9 right panel). This effect is stronger at recency positions due to the buffer. When the isolate is at the first position on the other hand, it is remember less frequently than its neighbor from position 2, or than the same word in a single-category list.

To summarize, the model predicts that at the beginning of a list, the probability to recall the critical word when presented within an isolate-list (Von Restorff condition) will be lower than the probability to recall the same word within a same-category list (since it is still not distinctive or surprising). Toward the end of the list this is expected to be inverted: the probability to recall the critical word within an isolate-list (Von Restorff condition) is higher than the probability to recall the same word in a same-category list. An experiment was constructed to test this hypothesis and simulation result.

## Experiment 2: memory for semantic isolates in IFR

The Von Restorff effect has been studied in the semantic domain by Hunt and Lamb (2001) and by Fabiani and Donchin (1995). However, little attention was given to the serial position of the isolate word within the list. The current experiment was constructed to further investigate the relationship between novelty and serial position as found in the previous experiment and model. An additional goal is to verify the model's prediction for comparison of the novelty effect in different serial positions to two possible baselines: a list of unrelated words, and list of related (same-category) words.

Therefore, two control conditions were included in the current semantic-isolates free recall experiment: a semantically related list and a semantically unrelated list. In order to compare the effect of the different types of lists as accurately as possible without confounds (due to the specific word chosen) the same critical word was embedded in three types of word lists: (1) a different category list (isolate condition), (2) a same category list, and (3) a list of unrelated words. For example, to create an isolate list, a critical word “Hour” was presented within the “animal” list: “Bear, Pig, Elephant, Deer, Cat, Mouse, Cow, Tiger, Horse, Lion, Rat.” To create a “same” category list, the word “Hour” was presented within the “time units” list: “Year, Decade, Second, Day, Century, Week, Millisecond, Minute, Month, Nanosecond, Millennium.” To create an unrelated list, “Hour” was presented within a list of randomly selected words, such as: “Prospect, Velvet, Account, Advance, Madam, Payment, Hunter, Pursuit, Circle, Clothing, Safety.”

To avoid presenting a critical word twice to one person, this comparison was performed between participants for each critical word. However, each participant contributed to all conditions while counterbalancing conditions with critical words. In addition, the contribution of frontal mechanisms to this effect was investigated. In order to test this with healthy young adults, a correlational approach was adopted. As previously, all participants completed a fluid intelligence test (Cattell and Cattell, 1963), and correlations were analyzed between the Von Restorff variables and IQ. The following experiment was replicated twice, with small methodological changes.

### Method

#### Participants

***Experiment 2a***. Fifty-four participants, all native English speakers, participated in the experiment for a monetary reward of £7. Age ranged 18–66 years, with a mean of 30.

***Experiment 2b***. Forty-seven students, all native English speakers, participated for class credits. Age ranged 20–48, with a mean of 31.

#### Design

The experiment was a 3 × 3 within participants design (4 × 3 in Experiment 2b). The first factor was the position of the critical word in a 12 words list (positions 1, 5, and 9, position 7 was added in experiment 2b). The second factor was the type of the embedding list. Critical words were presented as part of lists of either: (i) words that are all taken from the same category, but different to the category of the critical word (isolate condition), (ii) other words from the same category, or (iii) a list of unrelated words. Combinations of critical words, serial positions and type of embedding lists were counterbalanced between participants. Memory for critical words was measured under these different manipulations. Correlations between the memory measures and participants' IQ were computed.

#### Materials

Thirty-two categories were chosen from the Van Overschelde et al. (2004) norms, by the following criteria: (i) more than 20 items included in the category^2^, (ii) categories with overlapping or related items were excluded. 32 categories only were found to suffice these criteria. A list of 12 words was constructed from each category. One category member was chosen to act as the critical word. In order to measure the isolation effect, a critical word “a” (from category “A”) was presented either: (i) within a list of words from a different category “B,” (ii) within a list of words from its own category “A” or (iii) within a list of unrelated words. The different types of lists will be referred to as an “isolate” list, a “same-category” list and an “unrelated” list.

Memory for the critical words was compared between these three conditions between participants, to avoid remembering the critical item (“a”) from an earlier list (see example above). Unrelated words were taken from the Toronto word pool (http://memory.psych.upenn.edu/wordpools/wordpool.txt) taking care of eliminating overlap with the same-category lists.

Twenty-four lists were created by dividing the categories to groups of four and using the fact that the comparison had to be between participants: if categories “A,” “B,” “C,” and “D” are the categories of this group, and “a,” “b,” and “c” are the critical words and “R” is a list of random unrelated words, then they were presented to three different participants as in Table 1. It can be seen that category “D” was “recycled” to serve as an embedding list for the three different critical words: “a,” “b,” and “c” (but each was only presented once to each participant).

**Table 1:** **Building three lists: “Von Restorff,” “same category,” and “unrelated” from four categories, “A,” “B,” “C,” and “D,” by presenting the same isolate to three different participants, within three types of lists**.

	**Participant 1**	**Participant 2**	**Participant 3**
List 1	Isolate list:	Same category:	Unrelated:
	Isolate “a” within list “D”	Isolate “a” within list “A”	Isolate “a” within list “R”
	(Da)	(Aa)	(Ra)
List 2	Unrelated:	Isolate list:	Same category:
	Isolate “b” within list “R”	Isolate “b” within list “D”	Isolate “b” within list “B”
	(Rb)	(Db)	(Bb)
List 3	Same category:	Unrelated:	Isolate list:
	Isolate “c” within list “C”	Isolate “c” within list “R”	Isolate “c” within list “D”
	(Cc)	(Rc)	(Dc)

The same critical word was presented within the three different types of lists (“isolate,” “same-category,” and “unrelated”) to different participants in order to control for the contribution of the specific word chosen. The critical word appeared in the 1st, 5th, or 9th serial position (and also 7th position in 2b). This was counterbalanced with the different lists between participants. The experiment was programmed using the Eprime environment for psychological testing (version 1.1). Participants of experiment 2a completed the test in addition the Cattell scale 3 test (Cattell and Cattell, 1963). IQ scores were calculated accordingly.

#### Procedure

Participants first performed the memory experiment; participants of experiment 2a also performed the standard Cattell IQ test. During the memory experiment, participants were presented with 24 lists of words. The entire session lasted about 45 min, with a short break given after 12 lists. Participants were allocated to 9 (12 in Experiment 2b) groups for counterbalancing: each critical word was presented to three participants: once in an isolate list, once in a “same-category” list and once in an “unrelated” list. In addition, this was crossed with three (four in 2b) different orders for the location of the critical word in the list. The 24 lists that were constructed for each participant were presented in a random order. Each list comprised of 12 words, presented sequentially for 1500 ms on a computer screen. The participants were asked to read these words out loud. After the last word of the list, a question mark appeared. The participants were instructed beforehand to start recalling words from the list upon appearance of the question mark. They were asked to report the words in any order that they like. In experiment 2a they were asked to say each word out loud as soon as they remembered it (standard free-recall instructions). They were given 1 min for recall. The words reported were recorded by the experimenter in the order of their recall.

In Experiment 2b the students performed the tasks in a computer lab as a group. To maintain silence, they were instructed to read the words subvocally instead of out loud and type their responses instead of saying them.

### Results

The probability of recall (and probability of first recall) was calculated for each participant, for every list type and serial position. Since all conditions were counterbalanced, scores from the different lists were collapsed.

#### Memory for categorized lists vs. memory for unrelated lists

In previous studies, probability to recall words from same-category lists was higher vs. unrelated lists. To verify this, the data from all the “same” and “unrelated” lists were collapsed over the location of the critical word, as it does not make a difference in these lists which in not contain an isolate.

A 2 × 12 within participants ANOVA of list type (“same” and “unrelated”) vs. serial position (1–12) revealed significant main effects and interaction.

***Experiment 2a***. Main effect of list type: *F*_(1, 53)_ = 301.849, *p* < 0.001, MSe = 0.00001308, main effect of serial position: *F*_(11, 583)_ = 73.053, *p* < 0.001, MSe = 0.00001075, interaction between list type and serial position: *F*_(11, 583)_ = 12.618, *p* < 0.001, MSe = 0.000007934.

***Experiment 2b***. Main effect of list type: *F*_(1, 46)_ = 204.235, *p* < 0.001, MSe = 0.0000559, main effect of serial position: *F*_(11, 506)_ = 22.279, *p* < 0.001, MSe = 0.0000328, interaction between list type and serial position: *F*_(11, 506)_ = 4.940, *p* < 0.001, MSe = 0.0000225.

**Figure 10:**
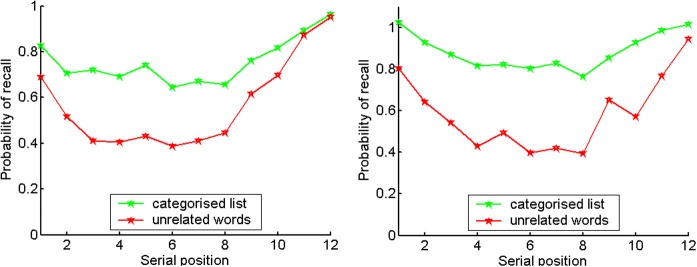
**Probabilities of recall for same-category (green) and unrelated lists (red)**. Right: Experiment 2a, left: Experiment 2b.

To conclude, there is significantly higher probability to remember words from a same-category list, and this difference is larger at middle list positions (see Figure 10).

#### Von restorff effects

Next, memory for critical words was compared between the different types of lists.

***Experiment 2a***. The probabilities to remember the critical words in the 1st, 5th, and 9th serial position when embedded in: an isolate list, a same-category list or a list of unrelated words, are presented in Table 2.

**Table 2:**
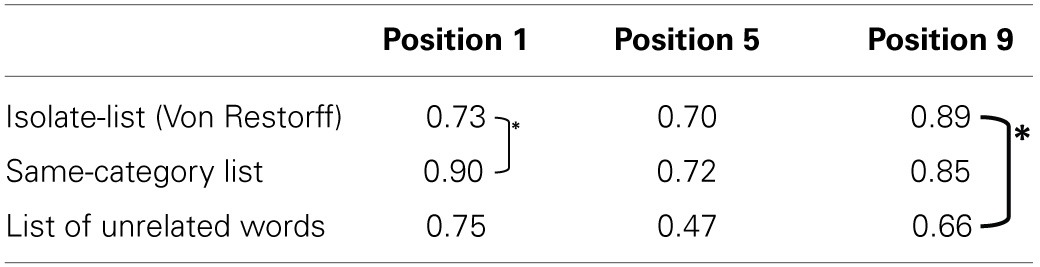
**Probability of recall for the critical word, within the different embedding lists**.

*Significant t-tests are marked with a star, and the condition with highest probability of recall for each serial position is highlighted (Experiment 2a)*.

***Experiment 2b***. The probability to remember critical words in the 1st, 5th, 7th, and 9th serial positions when embedded in an isolate-list in a same-category list and a list of unrelated words, is presented in Table 3.

**Table 3:**
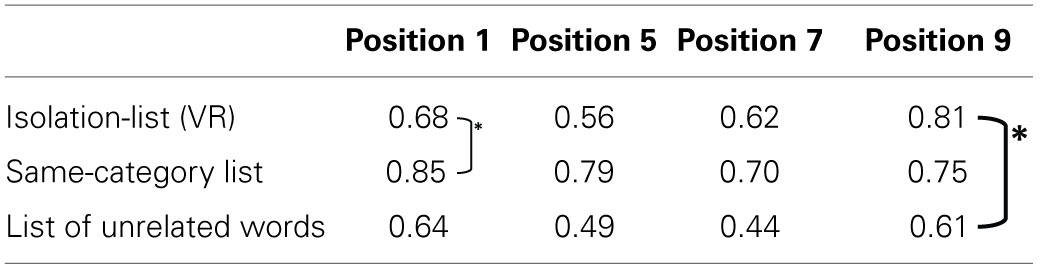
**Probability of recall for the critical item only, in the different embedding lists**.

*Significant t-tests are marked with a star, and the condition with highest probability of recall for each serial position is highlighted (Experiment 2b)*.

**Figure 11:**
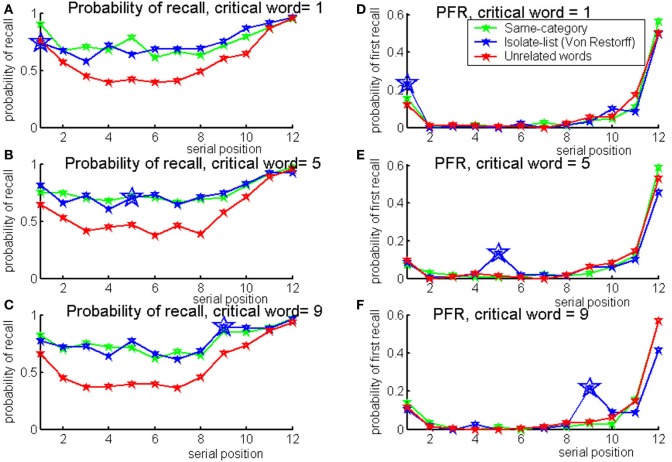
**Probability of recall (left) and probability of first recall (right) for lists with critical words at positions: 1 (top), 5 (middle), and 9 (bottom)**. In all panels, same-category lists are illustrated in green, isolate (Von Restorff) lists are illustrated in blue and unrelated lists are illustrated in red. Larger markers were used for the critical word in an isolate-list (Experiment 2a).

**Figure 12:**
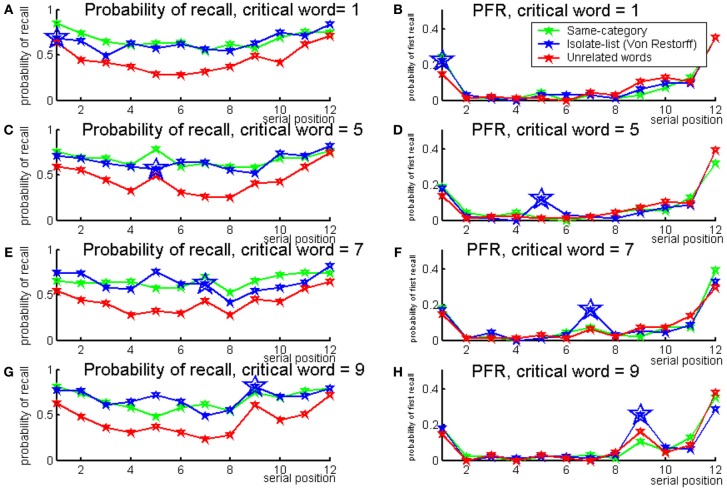
**Probability of recall (right) and probability of first recall (left) for lists with critical words at positions: 1 (top), 5 (2nd row), 7 (3rd row), and 9 (bottom)**. In all panels, same-category lists are in green, isolate (Von Restorff) lists are in blue and unrelated lists are in red. Larger markers were used for the critical word in an isolate-list (Experiment 2b).

The results indicate that at the first serial position, the probability to recall the critical word in an isolate-list is as low as in an unrelated list. At further serial positions, the critical word has an advantage in an isolate-list over the memory of the unrelated list. Probability to recall the critical word in an isolate-list is even higher than the same-category list for serial position number 9. The full serial position curves are presented in Figures 11, 12.

***Experiment 2a***. A 3 × 3 within participants ANOVA of list type and the serial position of the critical word, revealed a highly significant main effect of list type: *F*_(2, 106)_ = 20.458, MSe = 0.008235, *p* < 0.0001, highly significant main effect of critical word serial position: *F*_(2, 106)_ = 23.926, MSe = 0.006552, *p* < 0.0001, and most importantly, a highly significant interaction between the two factors: *F*_(4, 212)_ = 5.880, MSe = 0.005438, *p* < 0.0001.

The interaction was followed by planned *t*-tests: (i) at serial position 1 between the isolate and the same-category list conditions, (ii) at serial position 9, between the isolate and the unrelated words conditions and, (iii) between the isolate and the same-category list conditions. As predicted, in the first serial position memory for the critical word was lower in an isolate-list than in a same-category list: *t*_(53)_ = 3.6, *p* < 0.001; while in the 9th serial position memory for the critical word was higher in an isolate-list list than in an unrelated list: *t*_(53)_ = 3.97, *p* < 0.001. The isolate advantage over the same-category list at serial position 9 did not reach significance: *t*_(53)_ = 0.92, NS.

***Experiment 2b***. A 3 × 4 within participants ANOVA of list type and the serial position of the critical word revealed a highly significant main effect of list type: *F*_(2, 92)_ = 18.354, MSe = 0.134, *p* < 0.0001, and a highly significant main effect of critical word serial position: *F*_(3, 138)_ = 5.484, MSe = 0.132, *p* < 0.001. The interaction between the two factors failed to reach significance for this experiment, but shows a trend similar to the previous experiment: *F*_(6, 276)_ = 1.996, MSe = 0.105, *p* = 0.66.

Planned *t*-tests were performed: (i) between the isolate-list and the same-category list conditions at serial position 1, and (ii) between the Von Restorff to the unrelated words conditions at serial position number 9. In the first serial position, memory for the critical word was lower in an isolate-list than in a same-category list: *t*_(46)_ = 2.8, *p* < 0.01; while in the 9th serial position, memory for the critical word was higher in an isolate-list than in an unrelated list: *t*_(46)_ = 3.00, *p* < 0.005.

Following, the probabilities of first recall were analyzed (Howard and Kahana, 1999). The probabilities for the critical word in the different types of lists and serial positions in the list, are presented in Tables 4, 5.

**Table 4:** **Probability of first recall for the critical word only in different embedding lists (Experiment 2a)**.

	**Position 1**	**Position 5**	**Position 9**
Isolate-list (Von Restorff)	0.23	0.13	0.21
Same-category list	0.15	0.01	0.03
List of unrelated words	0.12	0.01	0.04

**Table 5:** **Probability of first recall for the isolate item only in different embedding lists (Experiment 2b)**.

	**Position 1**	**Position 5**	**Position 7**	**Position 9**
Isolate-list (VR)	0.22	0.12	0.17	0.26
Same-category list	0.25	0.01	0.07	0.10
List of unrelated words	0.15	0.01	0.06	0.16

The probability of first recall for all serial positions is presented in Figures 11, 12. It can be seen that the critical words have higher probability to be retrieved first when presented in an isolate-list.

***Experiment 2a***. This was verified using a 3 × 3 ANOVA of list type and serial position of the critical word, which revealed a main effect of list type: *F*_(2, 106)_ = 18.576, *p* < 0.001, MSe = 0.0050608.

***Experiment 2b***. This was verified using a 3 × 4 ANOVA of list type and serial position of the critical item, which revealed a main effect of list type: *F*_(2, 92)_ = 8.478, *p* < 0.001, MSe = 0.005966.

These results show that participants tend to start recall from the semantic isolates, especially for later serial positions. Therefore, it is likely that isolate words have a higher probability to stay active in a short term memory buffer.

#### Correlations with IQ

In Experiment 2a only, IQ scores were calculated for all participants based on their answers to the Cattell test. The average IQ for the sample is 115 (standard deviation of 16), which is higher than the general population average and could be explained by the large proportion of university students in the sample. For each participant and serial position, the number of times the critical word was recalled in a same-category list was subtracted from the number of times it was remembered in an isolate-list. Correlations between IQ and memory for the critical word in an isolate-list, in comparison to its memory in a same-category list is found for serial positions 1: *r*_(52)_ = 0.35, *p* < 0.01 and 5: *r*_(52)_ = 0.44, *p* < 0.001. For serial position 9 the correlation is only approaching significance: *r*_(52)_ = 0.244, *p* = 0.07.

### Summary

In both experiments we found that the advantage of semantic isolates in IFR is more prominent the later they appear in the list. At the first serial position, the Von Restorff effect was in fact reversed as the same-category words had their usual advantage in recall and overpowered the novelty advantage of the isolate. Memory for the isolate item at the first serial position was as low as in a control list of unrelated words. At serial position five, there was still no advantage for the isolate word. Its probability of recall was similar to the rest of the list and to the probability to be recalled in a list of its own category. At serial position 9 there was an advantage for the isolate word (the classic Von Restorff effect). In addition, isolate items had a higher probability to be recalled first, especially when in later serial positions.

We replicated the correlation result of Experiment 1. The magnitude of the Von Restorff effect was correlated with IQ, supporting the hypothesis that the distinctiveness mechanism is mediated by the PFC.

## General discussion

We have presented a combined experimental and computational investigation of the effects of semantic relations between words in IFR, and their mediation by frontal type mechanisms. First, among a set of memory variables, we found that the Von Restorff and semantic clustering showed the highest and most robust correlations with fluid intelligence, as measured by the Cattell test. This is consistent with previous literature which associated fluid intelligence (Duncan et al., 1995, 1996, 2000), semantic clustering (Baldo et al., 2002) and the processing of isolates (Fabiani and Donchin, 1995; Ranganath and Rainer, 2003; Kishiyama et al., 2009; Løvstad et al., 2012) with frontal mechanisms. The latter is indicated by reduced electrophysiological novelty-responses (P300) and novelty advantage in recognition memory for frontal patients (Kishiyama et al., 2009; Løvstad et al., 2012). In combination with these findings and with clustering deficits in the CVLT-task for frontal patients, the correlations above have motivated our focus on semantic clustering and the Von Restorff effect in the model we have developed.

The *CAN* model is based on a previous dual-store framework that was previously applied to account for serial position effects in IFR (Davelaar et al., 2005, 2006). The framework assumes that serial position effects in IFR are due to retrieval from episodic memory and from an activation buffer. Importantly, it combines a gradually changing temporal context representation which has been shown to account for recency effects in LTM (see Howard and Kahana, 2002), and an activation buffer that captures recency effects in STM. In contrast to single store models such as TCM, that assume that all recency effects are due to a changing context representation, the hybrid model is able to account for double dissociations between short- and long-term recency (Davelaar et al., 2005, 2006). In addition, the model predicted that increasing the presentation rate will bias the serial position function toward primacy effects, a prediction that counters all recency-based memory models and is explained by the dynamic nature of the activation buffer. It should be noted that changing-context models are essentially nested within the hybrid model. The added explanatory power of the activation buffer lends credence to its inclusion in the larger framework. Here, we augmented this dual-store, activation-context model with a number of novel components (the categorization and the novelty/distinctiveness mechanisms) which we associate with the PFC. The model reproduces serial position functions and accounts for the semantic clustering deficit of frontal patients in IFR. Moreover, it makes specific predictions for the dependency of the Von Restorff effect on the serial position of the isolate in the list.

These predictions were confirmed in two experiments. Isolates are remembered less well (compared with same category words) at the first serial position, while they are remembered as well as non-isolates in the middle of the list. Toward the end of the list they are better remembered. The probability of first recall is also higher for the isolates at middle and late serial positions, suggesting greater accessibility to these items at the beginning of the retrieval phase. The dependency of the isolate effect on the serial position within the list may seem to contradict earlier findings of a lack of serial position effect for the isolates in serial ordering tasks (McLaughlin, 1966; Bone and Goulet, 1968; Kelley and Nairne, 2001). Several differences exist between our experiments and those earlier reports. First, we used free recall compared to serial ordering: it is possible that the requirement for serial ordering tasks masks the subtle advantage for the isolate which is observable when recall is constraint-free. Second, most of the Von Restorff studies used isolates that were defined on perceptual dimensions: it is therefore possible that surface level features are insensitive to serial position effects, whereas deeper semantic processing requires more time to build up expectations, resulting in serial position effects.

Our model predicts frontal patients perform poorly on memory for isolate items. Indirect evidence from studies with frontal patients support this hypothesis (such as reduced P300 novelty signal, Daffner et al., 2000; Løvstad et al., 2012, however, a Von Restorff experiment using semantic isolates with frontal patients would be essential to confirm this prediction. Below we discuss the critical model properties that allow it to account for these results, and we then discuss its limitations and relation to other memory models that include frontal mechanisms.

### The *CAN*-model

The model successfully simulated CVLT and the Von Restorff effect. During encoding, the model associates similar items with the same categorization unit. At retrieval, activating this unit leads to semantic clustering as seen in the CVLT simulation. The isolation effect is captured by the model through the use of a novelty detection process that enhances the episodic learning of the isolate. Moreover, these mechanisms interact with the activation buffer to enable its functioning and account for the data.

In *CAN*, recall begins with the items that are active in short-term memory (Atkinson and Shiffrin, 1968; Davelaar et al., 2005). Both the probability of recall and the probability of first recall of isolates can be accounted for by a short-term memory process (activation buffer). Distinctive items which receive an additional top-down attentional boost survive the buffer competition for longer (see Figure 19 in Davelaar et al., 2005). The lower recall of isolates in earlier serial positions is probably also a result of the advantage of the same-category words, as they are better maintained in the buffer in the absence of top-down modulation. In addition, the buffer model accounts for the stronger VR-effect at the end of the list.

The activation buffer plays another major role in the CAN model, as it enhances the activation of semantic features that are shared between words which are co-active in the buffer. This allows these common feature units to connect online with the categorization units in the PFC. As this process is dependent on buffer co-activation, it predicts that semantic clustering would be stronger when the category words are contiguous (buffer co-active) rather than distant (i.e., separated by other words, and thus non-co-active) (Glanzer, 1969; Haarmann and Usher, 2001).

### Model limitations

Despite the model's ability to account for semantic effects in free recall that are mediated by frontal processes, the model has a number of limitations. First, we had to artificially limit the enhancement of input and learning due to “novelty” to the current item. Since the activations in all layers take time to update with the presentation of a new item, the difference between the predicted and the detected activations in the semantic features layer (which produces the “surprise” signal) can stay above the threshold longer than the novel item presentation. This can result in enhanced activation and learning of the item next in the list. Such effects do not exist in the experimental results, as the items following an isolate are not better remembered.

A second challenge in the novelty-mechanism, is a tendency for over-sensitivity. A “surprise” signal is generated when the difference between the predicted and the detected activations in the semantic features layer crosses a threshold. For all simulations, the same threshold was used. However, it proved difficult to set the threshold so the “surprise” would be generated for a semantic isolate, but not for a random item in an unrelated list. In some cases, the difference between two successive unrelated items can be as big as the difference between a semantic isolate and the category items, resulting in an irrelevant “surprise” signal. These irrelevant “surprise” signals have an undesirable effect on the encoding of unrelated lists, since the encoding of some items is enhanced, which leads to a difficulty in retrieving the other items. Essentially, this is a signal to noise problem, in which the signal should be detected while taking into account the magnitude of the noise. One approach to solving such a problem would be to adjust the difference between the predicted and the detected activations, using the variance of the activation in a certain time window:

|F(y)−a|/[var(F(B))+1]

In a Von-Restorff list, the variance of the semantic features layer prior to the presentation of the isolate would be small, as all items activate the same category features. Therefore, the correction factor [**var(F(B)) + 1**]] would have a minimal effect. In contrast, in an unrelated list, the variance of the activation would be larger, leading to a larger correction factor and a smaller value for the “surprise” term. Implementing this idea would require a neural network which computes variance online.

Finally, the CAN model is a model of free recall that extends over short time period. The model has a single context unit and thus is unable to capture such benchmark data as the long-term recency effect (Bjork and Whitten, 1974) and contiguity effects (Howard and Kahana, 1999) in continuous distractor free recall. Models that are able to capture such findings require a changing context representation (Howard and Kahana, 2002; Davelaar et al., 2005). Although augmenting the CAN model with a distributed changing context representation is an important focus for the future, we do not know whether isolation effects are invariant to temporal scale and whether the PFC mechanisms investigated here operate across longer time scales.

### Comparison with other models

The model shares some of the principles with Grossberg's ART model (1978a,b), in which bottom-up activated patterns are clustered into categories based on similarities in feature space. A category unit provides top-down bias, forming a self-stabilizing loop with the presented feature vector. When a feature is presented that is sufficiently different from the top-down expectations produced by the category unit, the model recruits a new unit to form a new category. Hence, ART has the ability to categorize similar items and to detect items that are sufficiently novel. This particular model, however, has not been applied to account for serial position functions in IFR. Although the related model LIST PARSE was able to capture general IFR data (Grossberg and Pearson, 2008), it did not address the semantic clustering and Von Restorff effects in IFR. Only few models have been developed to account for serial position functions and semantic effects (such as clustering and isolates in VR-lists) in free recall, and very few address the role of the PFC in those processes. Three such models are discussed here.

The first one is a model that explicitly addresses the role of the frontal cortex in semantic clustering in the CVLT task, which was developed by Becker and Lim (2003), while the second one, CMR (the Context Maintenance and Retrieval model; Polyn and Kahana, 2008) is based on the influential temporal-context framework (Howard and Kahana, 2002) which accounts for free-recall data, but does not relate its components to the PFC. The third model, NICE (the Novelty-Induced Change in Episodic context model; Davelaar, 2013) is based on a model by Estes (1955), and addresses primacy and novelty effects in first recall probabilities.

In the Becker and Lim (2003) model, semantic representations are combined with contextual representations in an MTL component, which is similar to the non-PFC components of our models (but without the buffer). As in the CAN model, the MTL component is connected to a PFC layer whose role is to learn the semantic categories in the list and facilitate semantic clustering at recall. To do this the model by Becker and Lim assumes that the MTL-PFC connections are modified during the simulation by reinforcement learning. During retrieval, successful recalls produce a reward signal, while intrusions and repetitions produce a punishment signal. Using this mechanism, the model spontaneously develops semantic clustering strategies. While the model shows similar improvement to human subjects during the five repetitions of the CVLT task, both in terms of probability of recall and semantic clustering, it does not perform as well as human subjects on the first presented list.

Thus, unlike the CAN model, the categorization requires multiple presentations of a list for recall. This raises the question whether the Becker-Lim model would be able to successfully recall (and categorize) a CVLT-like list of words at the first attempt, after it has been trained with another CVLT-like list.

The Becker-Lim model includes a neural network implementation of retrieval monitoring in order to decide if a retrieved word is a correct recall or an error (repetition or intrusion). The MTL module is probed with the retrieved word, and a similarity measure is produced between the word features and the MTL current state. If the similarity is too high, the word is classified as a repetition error, while if it is too low, the word is classified as an intrusion error. It is unclear, therefore, how the Becker-Lim model would treat the first recalled words which are usually the last in the list, as they are in danger to be incorrectly classified as repetition errors. Finally, the model focuses on the PFC layer, while other components are simplified. Therefore, it probably cannot predict basic findings in free recall, such as the serial position curve. It is also unclear if the Becker-Lim model could spontaneously develop a strategy to remember isolates in Von-Restorff lists.

Nevertheless, the Becker-Lim model is able to capture the increase in CVLT performance over several trials. Currently, the CAN model only simulates a single list, and a more thorough direct comparison with the Becker-Lim model will require the extending the CAN model to account for multi-trial free recall.

The second model discussed is a new variant of the TCM, the Context Maintenance and Retrieval model (CMR), which was developed by Polyn and Kahana (2008). Extending the previous TCM models (TCM and TCM-A; Howard and Kahana, 2002; Sederberg et al., 2008), CMR accounts for semantic clustering, as well as temporal and source clustering, and attribute an important role of the PFC to memory processes (Polyn and Kahana, 2008). The main difference between CMR and TCM is that the latter includes a fractionation of the context-representation into a temporal and a source context. Whereas words that are encoded successively share overlap in their temporal context, words that were processed in similar ways (e.g., orienting task) share source context. This allows the model to account for source clustering in addition to temporal clustering. Furthermore, CMR assumes that semantically related items have similar pre-experimental context representations; this is similar with our assumption that similar words have shared semantic representations. Semantic categorization arises since retrieval is guided by the context representation.

Unlike in the CAN model however, in CMR, semantic clustering happens exclusively during the retrieval stage. When an item is retrieved, its context is added to the current context vector, and therefore facilitates retrieval of a semantically related item through a pre-experimental connectivity matrix. There is evidence however, that semantic clustering does not happen solely during retrieval. For example, frontal patients benefit from encoding instructions (e.g., Incisa della Rocchetta and Milner, 1993; Ward, 2003), and activations are found in neuroimaging studies during the encoding stage of a categorized list.

The context layer of the CMR model has separate temporal and source features. When a change occurs in the source component (in Polyn and Kahana, 2008 this represents a shift in the encoding task), a “disruption” process takes place by presenting to the network a new item (which is not learned), while increasing the change rate of the whole layer. The “disruption” creates a large change in the existing temporal representation. In CMR, this enhances source clustering by weakening the contextual similarity between items from different sources. This “disruption” process is similar to the “surprise” mechanism of the model presented above. In the case of a Von Restorff list, an experimental hypothesis can be drawn that the “disruption” process would cause separate clusters for words which appear before and after the isolate. However, it is unclear if the “disruption” process could facilitate memory for semantic isolates.

Finally, a recent single-store model was able to capture Von Restorff effects and primacy effects in the first recalls by using only a single novelty-detection process (Davelaar, 2013). The Novelty-Induced Change in Episodic (NICE) context model is a distributed context model with binary elements. The model is assumed to reflect activation patterns in the medial-temporal lobe that support recall performance. At each time-step, the activation profile of the elements is updated according to a matrix of transition probabilities (see Estes, 1955; Mensink and Raaijmakers, 1988, for similar models). The NICE context model assumes however, that the transition probabilities are not fixed but are a function of the novelty of an item with increased novelty leading to faster contextual change. The precise calculation for novelty was assumed to come from outside the distributed context representation, and involves the current contents of a limited-capacity buffer.

The NICE context model provides a single process solution for the observation of primacy effects in the first recall probability functions by assuming that the very first item of the free recall list is novel. The current PFC model provides a mechanism through which novelty is calculated, and could be added to the NICE context model. Interestingly, the transitions in the NICE context model leads to more active elements being associated with a novel item. Thus, both the CAN model and the NICE context model predict stronger episodic traces for novel items. Adding the NICE context components to the CAN model will make the hybrid model able to account for primacy effects that are not due to rehearsal or buffer processes, and allow it to address Von Restorff effects in long-term memory paradigms, such as the continuous distractor task (Bjork and Whitten, 1974).

### Future directions

The model for the role of the PFC in free recall memory hereby presented can be further developed in a number of ways. In order to investigate the performance of the frontal mechanism in relative isolation, it was added to a simplified version of the buffer-activation model. The representation of context was limited to a single unit, or list context. Further investigations could integrate an elaborate distributed context (such as in the NICE context model) with PFC mechanisms (see also Elhalal and Usher, 2011). In addition, the novelty-detection mechanism can be developed, as previously discussed.

Experimental investigations may further examine our model prediction that clustering and VR-effects are contingent on STM co-activation. For example, we predict lower clustering and Von Restorff effects in the continuous distractor free recall paradigm, in which the memory items are not co-active in the STM-buffer. Finally, further testing of IFR with semantic isolated in needed in frontal populations.

## Conclusions

The Von Restorff paradigm was explored in this paper using both experiments and computational modeling. This has been a useful platform for investigating the effects of semantic relatedness and semantic isolation. A large body of evidence exists for the role of the frontal cortex in these functions, including the correlations presented between sensitivity to novelty and fluid intelligence. We have provided a computational framework within which dynamic processes mediated by the prefrontal cortex contributes to semantic clustering and to the Von Restorff effect in free recall.

### Conflict of interest statement

The authors declare that the research was conducted in the absence of any commercial or financial relationships that could be construed as a potential conflict of interest.

## Footnotes

^1^For example, in the “sleep” list, the words presented are all strong associations of “sleep,” which is not presented: “bed, rest, awake, tired, dream, wake, snooze, blanket, doze, slumber, snore, nap, peace, yawn, drowsy” (Roediger and McDermott, 1995).

^2^This criterion was employed to prevent participants from developing a strategy of merely guessing category members without actually remembering them from the list. For example, a list of simple colors would make a guessing strategy quite successful.
