# Supplementary material for: The role of the frontal cortex in memory: an investigation of the Von Restorff effect
Source: Front Hum Neurosci. 2014 Jun 27;8:410. doi: 10.3389/fnhum.2014.00410 (PMC4073090; doi:10.3389/fnhum.2014.00410)
Supplement: Supplementary file 1 [file DataSheet1.DOCX]

**Supplement**

*Experiment-1: additional data*

We examined the First recall probabilities in categorized lists for blocked vs non-blocked conditions, separated for High/Low IQ-groups.

Figure S1: First recall probabilities for blocked and non-blocked categorized lists in experiment 1, separated by high (blue) and low (red) fluid-IQ groups.

We observe that with the blocked lists, the high IQ participants show an increased tendency to start recall with the first item of the last category. This is consistent with our account of a novelty signal computed by frontal mechanism and assisting the recall of that item.

We also examined serial order effects by computing conditional probability for recall or lag-recency (Howard & Kahana, 2002), for categorized versus random list.


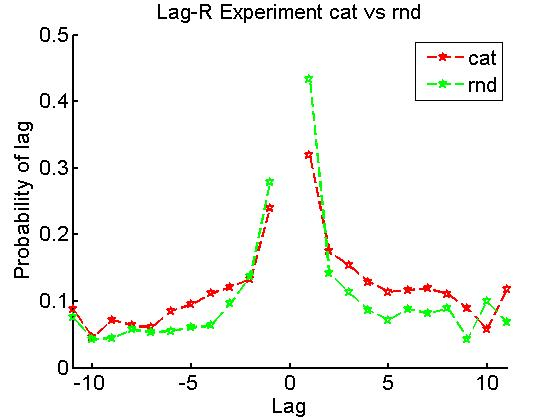


Figure S2: Lag-recency effects in experiment 1 for categorized lists (in red) and random lists (in green).

We observe typical lag-recency functions, which are more peaked in the random compared with the semantically related condition. This indicates that semantic relations provide an additional component to guide recall, on top of changing context mechanisms (cf Polyn, Erlikhman & Kahana, 2011).

*Details on Computer simulations*

***Parameter values***

Below are the parameter values used for the simulations ahead:

| Parameter | Value | Description |
| --- | --- | --- |
| **** | 0.9 | ** **time constant of the lexical, semantic and categorisation layers |
| **α** | 1.4 | the self excitation parameter of the lexical layer |
| **β_x_** | 0.18 | the global inhibition parameter of the lexical layer |
| **T** | 25 | number of time steps for a single item encoding |
| I | 0.35 to the current list item, and zero to all other items. | sensory input |
| **** | 0.006 | theparameter multiplying the activation spreading from the semantic features to the lexical layer |
| **β_y_** | 0.13 | the global inhibition parameter of the semantic features layer |
| **** | 0.7 | theparameter multiplying the activation spreading from the lexical layer to the semantic features layer |
| a(t=1) | 0.03 for all units | the initial value of the adaptation |
| **λ_a_** | 0.98 | time constant of the adaptation |
| **** | 14 | the parameter multiplying the adaptation term in the semantic features layer activation |
| **β_z_** | 14 | the parameter multiplying the adaptation term in the semantic features layer activation |
| **** | 0.7 | the parameter multiplying the activation sent from the semantic features layer to the categorisation layer |
|  | 0.3 | learning threshold for categorisation units: |
| **** | 7 | learning rate for the connections between the categorisation and the semantic layers |
|  | 0.1 | threshold for novelty detection: |
| I_s_ | 3.5 | sensory input, for items detected as novel |
| ****_s_ | 28 | learning rate for the connections between the categorisation and the semantic layers, for items detected as novel |
| TR | 150 | number of retrieval time steps |
|  | 0.12 | retrieval threshold |
| **μ_x_** | 0.001 | the parameter multiplying the activation sent from the context to the lexical layer at retrieval |
| **μ_y_** | 0.03 | the parameter multiplying the activation sent from the context to the semantic features layer at retrieval |
| **** | 1 | the parameter multiplying the activation sent from the categorisation layer to the semantic features layer at retrieval |
|  | -10 | reset value of retrieved items |
| **μ_z_** | 0.01 | the parameter multiplying the activation sent from the context to the categorisation layer at retrieval |
| **φ_ret_** | 0.05 | the parameter multiplying the activation sent from the semantic features layer to the categorisation layer, during retrieval |
|  | 0.02 | reset value of categorisation layer after 2 or more unsuccessful recalls |
| I_s_ | 3.5 | sensory input, for items detected as novel |

**CVLT-simulation details**

In the CVLT task, participants are asked to memorise a list of 16 words from 4 different categories which are presented in a random order. The CAN model is used to simulate the differences in semantic clustering between frontal patients and normal subjects.

The lexicon for this task is constructed of 4 categories. The first category is represented by semantic features 1-5, the second by 6-10, the third by 11-15 and the fourth by 16-20. 4 items are constructed of each category, with 5 common category features in addition to randomly selected specific item features. These 16 items are presented in a random order, with no two consecutive items from the same category, similar to the CVLT paradigm. Presentation order changes with every run of the simulation. For example see Figure S3. In this example items 2,8, 11 and 15 belong to the first category, items 4,6,10 and 13 belong to the second category, items 1,5,7 and 12 belong to the third category and items 3,9,14 and 16 belong to the fourth category.

The activation in the semantic features layer during encoding can be seen in Figure S4a. The features which represent the different categories become active in accordance to the times when the items receive input (with some delay due to the inhibition in this layer), while specific features can become active as well, due to the adaptation in this layer.


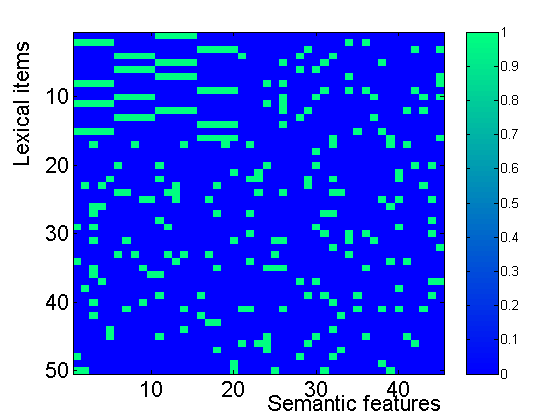


List items

Unstudied items

Figure S3: The connectivity matrix between the lexical items (vertical) and the semantic features (horizontal): W^xy^. The lexicon consists of 50 items. The first 16 items serve as list items and belong to 4 different categories. The first category is represented by semantic features 1-5, the second by 6-10, the third by 11-15 and the fourth by 16-20. Words are presented in a random order. (A connection exists where there is a value of 1 – green in the matrix. Values of 0 represent no connections and appear in blue).


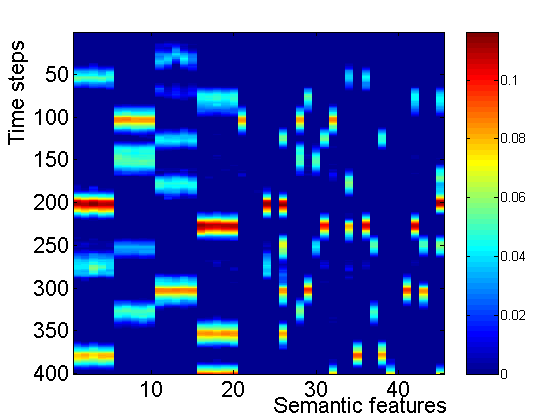

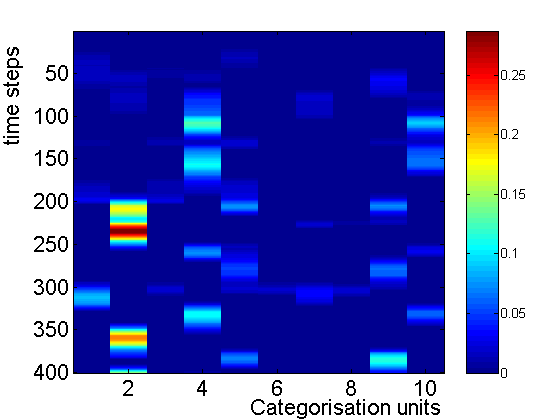


Figure S4: Activation in the semantic features (left) and the categorisation (right) layers during encoding in a CVLT simulation of the lexicon presented in Figure S3. Warmer colours represent higher activation. Each item is presented for 25 time steps, therefore encoding of 16 items takes 400 time steps.

The activation in the categorisation layer during encoding can be seen in Figure S4b. Categorisation units number 4,2,1,5, 9 and 10 become active at different intervals during list presentation.

In order to explore the learning that takes place between the categorisation layer and the semantic features layer, the connectivity matrix between them, before and after the encoding stage, is presented in Figure S5. It can be seen that the categorisation layer has learned the categories in the list (right side of Figure S5): The connections between categorisation units 5 and 9 and semantic features 1-5 (which represent the first category) became stronger. In the same way, categorisation units 4 and 10 became associated with the second category, categorisation unit 1 became associated with the third category (albeit weakly) and categorisation unit 2 became associated with the fourth category.


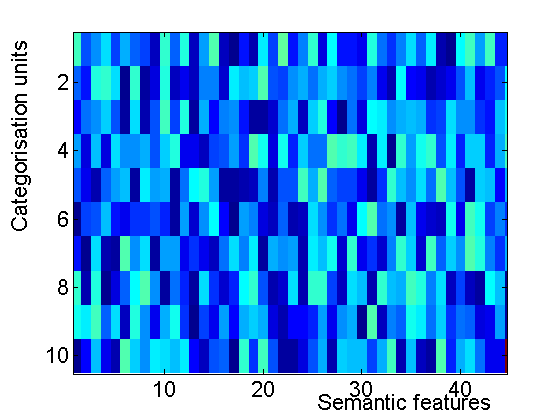

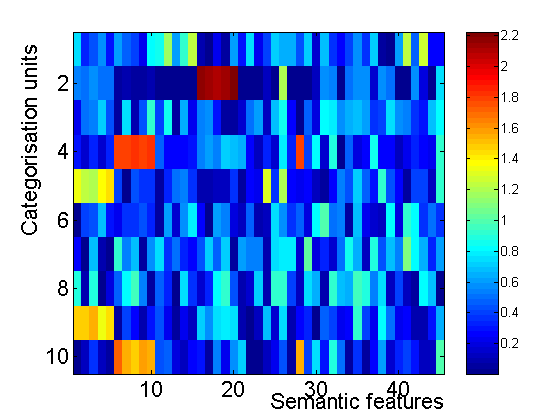


Figure S5: The connectivity matrix between the semantic features (horizontal) and categorisation layer (vertical), in the beginning of the simulation (left) and after encoding – learning the categories in the list (right). Warmer colours represent stronger connections. It can be seen that different categorisation units became associated with the four categories.

During retrieval, the active categorisation unit(s) send(s) activation to the features they are connected to, and therefore enhance the semantic clustering of the recalled items.

**Von Restorff simulation details**

The experimental results indicate that while isolate items have advantage in late serial positions, they are less well remembered when they appear in the beginning of the list. The CAN model is applied to simulating these data.

The lexicon for this task is constructed of 2 categories. All studied items except one belong to a single category, while the isolate item belongs to a different category. The isolate is simulated at three serial positions (see top panel of Figure S6). As before, the first category is represented by semantic features 1-5, and the second category is represented by semantic features 6-10. In addition, each item has randomly selected specific features.


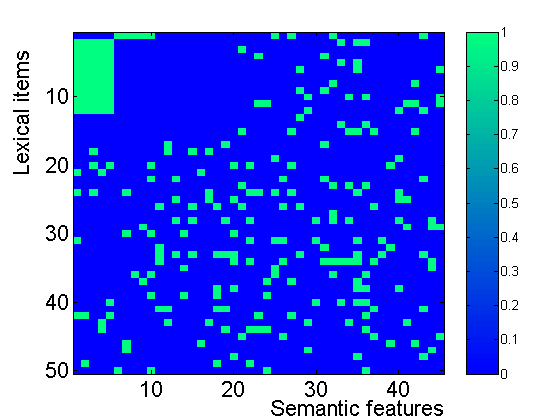

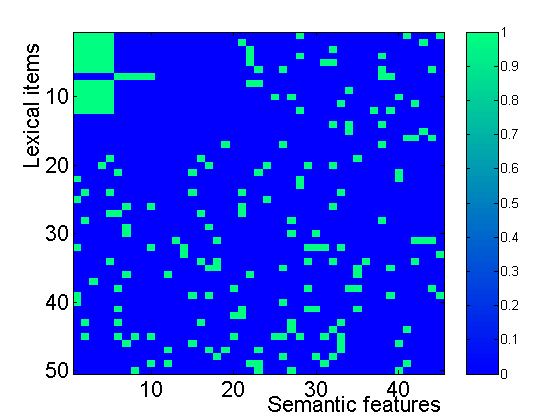

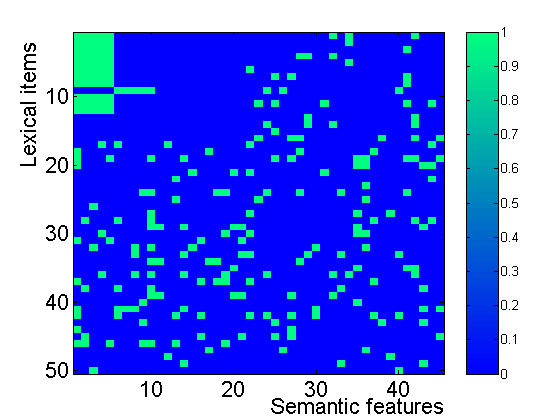


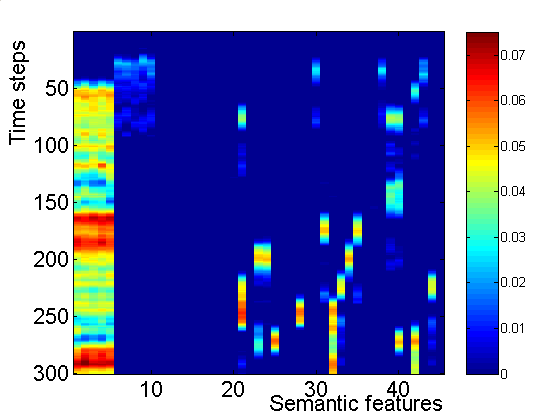

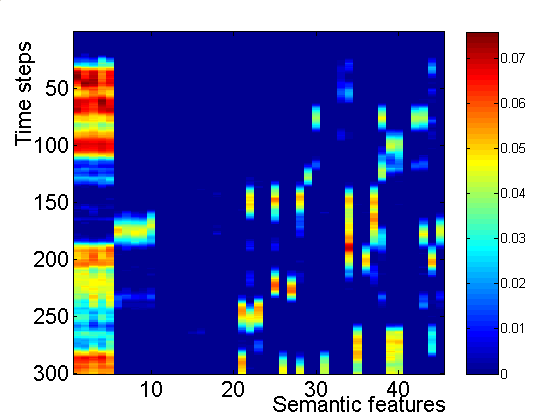

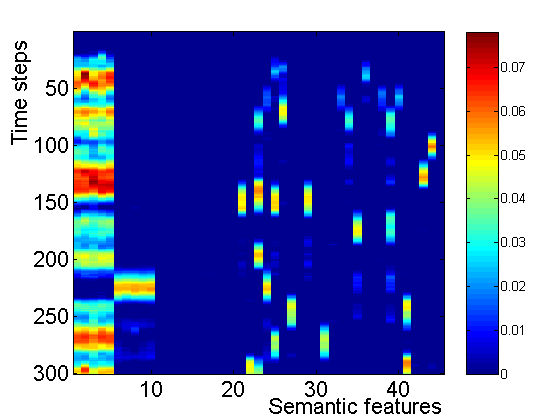


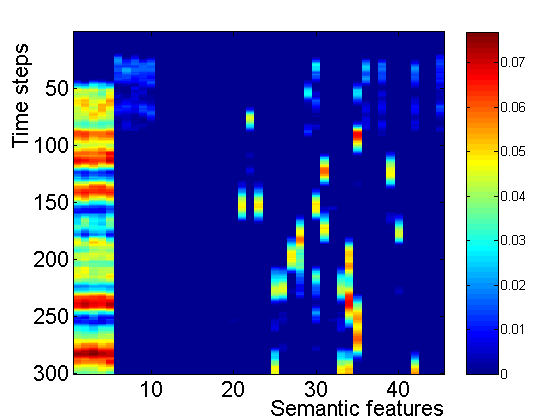

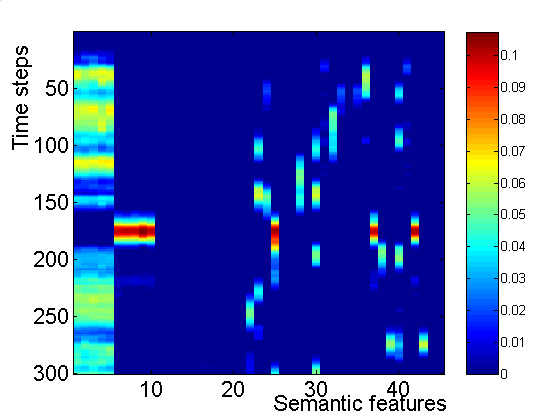

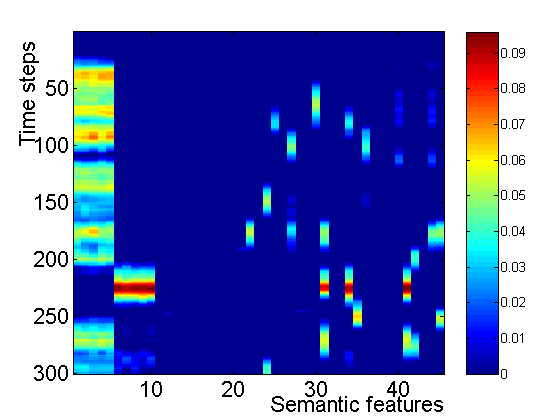


Figure S6: The semantic features layer in a Von Restorff simulation:

Top: The connectivity matrix between the lexical items (vertical) and the semantic features (horizontal): W^xy^. The lexicon consists of 50 words. The first 12 items, except one, belong to one category which is represented by semantic features 1-5. A single item (the isolate) belongs to a second category which is represented by semantic features 6-10. In the left panel the isolate appears in the first serial position, while in the middle panel it appears in the 7^th^ position and in the right panel in the 9^th^ position. Connections (value of one) appear in green, while value of zero (no connection) appears in blue.

Middle: the activation in the semantic features layer, in a simulation without the frontal mechanisms, of a Von-Restorff list. The isolate appears in serial position 1 (left) 7 (middle) and 9 (right). Warmer colours represent higher activations.

Bottom: same, but in a simulation which includes the frontal mechanism. It can be seen that isolates in positions 7 and 9 have higher activations than the category items, which is not the case in the middle panel.

The activation in the semantic features layer without the frontal mechanisms can be seen in Figure S6 (middle) for isolates at serial positions 1, 7 and 9 (as before).

In comparison, the activation in the semantic features layer with the addition of the frontal mechanisms can be seen in the bottom panel of Figure S6. The isolate in positions 7 and 9 activates the “surprise” mechanism of the model. The activation of the isolate’s semantic features is higher relative to the model without the frontal mechanisms, and therefore the isolate is better encoded.

One extra assumption was assumed to account for the peak in the first-recall probability at middle positions (Figure 9, middle-right; main text). We assumed that once an item is boosted by the novelty-surprise mechanism, its self-excitation buffer parameter, **α** is enhanced (e.g., as a result of neuromodulatory triggered learning). This allows isolate at middle position to be maintained longer in the buffer, in some cases be the first item to be recalled. Without this boost we find peaks for the isolate at middle and recency positions in total-recall, and for the first recall probability only at recency positions.
